# Clinical Success of Pulpotomies Using Intermediate Restorations and Preformed Metal Crowns in the Context of a Developing Country: A Retrospective Questionnaire-Based Investigation

**DOI:** 10.3390/clinpract14060203

**Published:** 2024-11-27

**Authors:** Ana Luisa Montero-Copoya, Norma Leticia Robles-Bermeo, Salvador Eduardo Lucas-Rincón, María de Lourdes Márquez-Corona, Saraí Carmina Guadarrama-Reyes, Nayeli Lovera-Rojas, Juan Fernando Casanova-Rosado, Juan José Villalobos-Rodelo, Mauricio Escoffié-Ramírez, Carlo Eduardo Medina-Solís

**Affiliations:** 1 Specialty Program in Pediatric Dentistry, Advanced Studies and Research Centre in Dentistry “Dr. Keisaburo Miyata”, School of Dentistry, Autonomous University of the State of Mexico, Toluca 50130, Mexico; almonteroc001@uaemex.mx; 2 Advanced Studies and Research Centre in Dentistry “Dr. Keisaburo Miyata”, School of Dentistry, Autonomous University of the State of Mexico, Toluca 50130, Mexico; scguadarramar@uaemex.mx (S.C.G.-R.); nloverar@uaemex.mx (N.L.-R.); 3 Academic Area of Dentistry of Health Sciences Institute, Autonomous University of Hidalgo State, Pachuca 42130, Mexico; salvador_lucas@uaeh.edu.mx (S.E.L.-R.); lmarquez@uaeh.edu.mx (M.d.L.M.-C.); 4 School of Dentistry, Autonomous University of Campeche, Campeche 24039, Mexico; jfcasano@uacam.mx; 5 School of Dentistry, Autonomous University of Sinaloa, Culiacan 80040, Mexico; juanvillalobos@uas.edu.mx; 6 School of Dentistry, Autonomous University of Yucatan, Mérida 97000, Mexico; mauricio.escoffie@correo.uady.mx

**Keywords:** oral health, pediatric dentistry, pulpotomies, IRM, metal crowns, developing country, Mexico

## Abstract

**Background:** Dental caries is one of the most prevalent dental illnesses in children. Untreated severe caries can damage teeth, requiring pulpotomy to save them. **Objective:** To evaluate the clinical success rate of treatments involving pulpotomies + IRM (Intermediate Restorative Material, composed of reinforced zinc oxide–eugenol polymers) + preformed metal crowns (PMCs) in primary teeth of children treated at a public university in Mexico. **Materials and Methods:** A cross-sectional ambispective study was conducted on children aged 10 years or younger, treated at a public university in Mexico. A total of 1281 medical records from February 2018 to June 2023 were reviewed, yielding a sample of 199 children treated with pulpotomy + IRM + PMC. Information was collected via telephone interviews with parents/caregivers to inquire about any symptoms following the pulpotomy and metal crown treatments. Success was defined as the absence of pain, inflammation, or infection. **Results:** During the study period, the number of teeth treated with pulpotomy + IRM + PMC was 414, with follow-ups ranging from 6 to 69 months. Most participants were girls (54.3%), while 45.7% were boys. Out of the 167 pulpotomy + IRM + PMC treatments included in the study, the clinical success rate was 98.2%. **Conclusions:** The clinical success rate of primary teeth treated with pulpotomy + IRM + PMCs was high, close to 100%, based on evidence and symptom reports from parents/caregivers. It would be beneficial to implement its use in public health institutions in countries such as Mexico.

## 1. Introduction

Dental caries is a progressive disease that begins with a white spot resulting from demineralization and is among the most common dental diseases in children. It impacts children’s quality of life by causing pain, sepsis, suffering, discomfort, difficulty eating and speaking, and loss of school time, among other issues [1,2,3]. Its etiology is multifactorial, but good oral hygiene habits, reduced consumption of fermentable carbohydrates, adequate fluoride exposure, and proper access to oral health services can help prevent this disease [4,5]. According to the Global Burden of Diseases, the age-standardized prevalence rate of untreated caries in primary teeth was 7.8% (573 million affected) [6]. The prevalence of dental caries in Mexican children is notably high, exceeding the global average. This high prevalence creates an urgent need for effective, accessible, and affordable treatment approaches that can be successfully implemented within the public healthcare system. National data indicate that approximately 50% of school-age children between 5 and 16 years old in Mexico present [7] or report having dental caries [8], making it a public health problem due to its high prevalence and incidence. If deep caries in primary teeth is not treated promptly, it can lead to defects in dental tissue, pulp injuries, and periapical infections, ultimately resulting in the early loss of primary teeth [9]. Therefore, effective treatments for severe dental caries are a priority for developing countries such as Mexico, where unfavorable oral health scenarios present challenges.

Existing literature highlights that socioeconomic disparities significantly influence access to preventive and restorative dental care, particularly in developing countries. Children from low-income families often experience delayed or inadequate treatment for dental caries, leading to more severe disease and increased risk of pulpal complications and tooth loss [10,11,12,13]. Resource limitations in many developing countries present challenges in providing timely and effective dental care, particularly for managing severe caries in primary teeth. Access to specialized materials and trained professionals may be limited, impacting treatment outcomes [11,14,15,16].

Pulpal pathologies in children are common, and the treatment of choice is pulp therapy. The aim of pulp therapy is to maintain primary teeth in the mouth until their natural exfoliation, preventing premature losses that can cause chewing and speech problems, loss of space, and malocclusions [17]. Pulpotomy in primary dentition is one of the most common treatments in pediatric dentistry, with multiple material options available for placement after pulp amputation, including ferric sulfate, formocresol, glutaraldehyde, calcium hydroxide, mineral trioxide aggregate (MTA), Biodentine, and Intermediate Restorative Material (IRM) [18,19,20,21]. IRM helps preserve pulp vitality, maintains sealing, prevents leakage, and is a biocompatible material [22]. Pulpotomy aims to preserve the tooth by treating it and subsequently rehabilitating it through a restoration that has a high survival success rate, fulfilling the objective of pulp therapy. Preformed metal crowns are restorations that replace the coronal portion of the damaged tooth and are the most common way to repair and maintain the remaining dental tissue [23]. They are widely used after pulp therapy in primary teeth, demonstrating high durability and resistance, ensuring they remain in the mouth long enough until the exfoliation of the dental organ and providing ease of handling and technique [24,25].

Historically, various materials have been used in pulpotomies, each with its advantages and disadvantages. Formocresol, while once widely used, has been largely replaced due to its toxicity concerns. Other popular materials that have been used over time with acceptable results include ferric sulfate or sodium hypochlorite. Current approaches frequently utilize calcium hydroxide, MTA, or Biodentine, all of which offer superior biocompatibility [26,27,28]. The use of Intermediate Restorative Material (IRM) as an intermediate restoration before final crown placement represents a cost-effective strategy commonly employed in resource-constrained environments [29,30,31]. Preformed metal crowns (PMCs) are favored for restoring primary teeth after pulpotomy due to their exceptional durability, ease of application, and proven longevity in clinical settings. Studies indicate a significant increase in long-term treatment success when PMCs are used [32,33].

While several public health initiatives aim to address dental caries in Mexico, challenges persist due to limited resources and unequal access to care [7,8,34,35,36,37]. Studies in similar developing nation contexts have shown similar trends of poor access to care, emphasizing the need for readily available and cost-effective solutions. The objective of this study was to evaluate the clinical success rate of treatments involving pulpotomies + IRM (composed of reinforced zinc oxide–eugenol polymers) + PMC in primary teeth of children treated at a public university in Mexico.

## 2. Materials and Methods

### 2.1. Study Design and Sample Selection

A clinical cross-sectional ambispective study was conducted, reviewing medical records of children who attended the Pediatric Dentistry Specialty Clinic at the Autonomous University of the State of Mexico (Toluca, Mexico) from February 2018 to June 2023. The sample size calculation was performed using the following parameters: a total population of 1300, a confidence level of 95%, a precision of 4%, a proportion (approximate value of the clinical success rate of treatments involving pulpotomies) of 90%, and an expected proportion of losses of 5%, resulting in 195 subjects. A total of 1281 medical records were reviewed, which had to meet the following inclusion criteria: (1) Complete medical records, (2) Medical records of children aged 1 to 10 years, (3) Both sexes, (4) Primary molars diagnosed with deep caries based on clinical and radiographic examinations, (5) Medical records with at least one pulpotomy treated with IRM and restored with a preformed metal crown, (6) Medical records documenting pulpotomy and crown treatments with a minimum follow-up of 6 months. The exclusion criteria were as follows: (1) Parents/caregivers unwilling to provide information, (2) Parents/caregivers who could not be contacted. After applying the inclusion and exclusion criteria, the final sample consisted of 199 medical records.

### 2.2. Setting and Context

The university provides specialized services at low cost to the general population of the region of all socioeconomic levels, although it is mainly attended by those of medium and low socioeconomic status. In the clinics of the pediatric dentistry service, the clinical histories and treatments are performed by students of the Pediatric Dentistry specialty of the Autonomous University of the State of Mexico. At the beginning of the semester, they are trained and standardized in the criteria used, and they are also familiarized with the sections and form of the proper registration of clinical histories.

### 2.3. Clinical Procedure

In general, the standardized clinical procedure for performing pulpotomy and placing the stainless-steel crown at the university clinic, which is performed under absolute isolation and infection control protocols throughout the procedure, is as follows:

#### 2.3.1. Clinical Procedure for Pulpotomies

1. Diagnosis and anesthesia: A thorough clinical examination is performed, including caries detection and radiographic evaluation (periapical radiograph). Local anesthesia is administered as necessary (2% Lidocaine with 1:100,000 epinephrine). Isolation is performed with a rubber dam to prevent contamination.
2. Caries removal: Careful removal of carious dentin is performed using sterile high-speed burs and copious irrigation with water to reach the pulp chamber.
3. Pulp amputation: Once the pulp chamber is accessed, the coronal pulp is carefully removed using a sterile excavator. Hemostasis is achieved using various methods such as gently blotting with absorbent tips or sterile cotton balls. Pulpal bleeding is completely controlled before proceeding with the aid of copious irrigation with sterile saline solution and by applying pressure with sterile cotton swabs to produce hemostasis.
4. IRM Placement: After adequate hemostasis, a temporary filling material, commonly Intermediate Restorative Material (IRM), is carefully placed to seal the pulp chamber. This step serves as an intermediate restoration. It is verified that the IRM does not protrude beyond the occlusal surface.

The use of other filling materials such as mineral trioxide aggregate (MTA), calcium silicate (Biodentine), or other biocompatible materials over the amputated pulp to protect the remaining pulp tissue is also considered. These materials are used if the patient can afford the cost of treatment using these materials.

#### 2.3.2. Clinical Procedure for Crown Placement

1. Tooth Preparation: The tooth is prepared for crown placement using appropriately sized finishing burs. Any excess IRM is removed, and the surface is smooth and clean.
2. Crown Selection: A properly fitting stainless steel crown is selected based on the size of the tooth or, if the crown to be rehabilitated is too damaged, using the tooth on the opposite side as a reference. The crown fits perfectly without excessive pressure.
3. The crown is tested to check the occlusal and gingival fit of the crown. That is, the patient’s occlusal relationship is reconstructed, and the length of the crown is checked to ensure it is 0.5 to 1 mm below the free edge of the gum. If this is not the case, the crown is trimmed so that it does not cause ischemia in the surrounding gum.
4. Once trimmed, the crown must be contoured in the cervical third with pliers for this purpose (Model 678-221, Hu-Friedy, HYGENIC, Johnson; Chicago, IL, USA), to fit the cervical portion of the metal crown to the cervical margin of the tooth.
5. Finishing and polishing: Once the adaptation of the crown to the tooth and the patient’s occlusion is complete, the previously trimmed cervical portion is polished by adapting the length of the metal crown with rubber tips or cups.
6. It is recommended to take a periapical X-ray to check the adaptation of the crown to the tooth, in a continuous finish and without spaces between the tooth and the restoration. The X-ray will allow the correction of the adaptation, if necessary, prior to cementation.
7. Cementation: The selected crown is cemented using an appropriate cementation agent (for example, glass ionomer cement). Excess cement is carefully removed.
8. Postoperative instructions: Parents or guardians are provided with detailed instructions regarding postoperative care, including oral hygiene, dietary advice, and follow-up appointments.
9. Postoperative X-ray: A control X-ray is taken at the end of treatment.

### 2.4. Data Collection

The examiner collected information from medical records (retrospective part) and from interviews (prospective part) directed to parents or caregivers of the selected children. The interviews were conducted via telephone. A database was created using Microsoft Excel to analyze the collected information. The data included age at the time of treatment, sex, number of lesions requiring treatment (pulpotomy + IRM + PMC), presence or absence of the dental organ, presence or absence of pain in the treated tooth, presence or absence of inflammation, and presence or absence of a fistula. Clinical success was defined as the absence of pain, inflammation, fistula, and/or infection, and was the dependent variable.

Parents/guardians were asked a few questions to determine the success of the treatment, focusing on the absence of symptoms:

1. Since the procedure, has your child experienced pain or persistent sensitivity in the treated tooth?
2. Has the treated tooth area shown signs of swelling, redness, or pus?
3. Did the crown fall off? If so, when did this happen and what were the associated symptoms?

### 2.5. Statistical Analysis

Descriptive data were obtained in the statistical analysis, reporting measures of central tendency and dispersion. Due to the high success rate observed, and therefore a comparison category with very low frequency, analysis by age and sex could not be performed. Therefore, the study will have that limitation in the discussion of the results.

### 2.6. Ethical Aspects

The research project was approved by the ethics committee of the Faculty of Dentistry at the Autonomous University of the State of Mexico, with registration code CEICIEAO-2023-015.

## 3. Results

A total of 1281 medical records were reviewed, of which 199 were selected that met the inclusion and exclusion criteria. In total, 414 dental organs were recorded with pulpotomy + IRM + PMC treatment. The response rate was 40.70% (81/199 children), with 167 teeth included in the analysis.

The analyses presented below are from children who participated in the study. Regarding sex distribution, it was observed that the majority were female, representing 54.3% (n = 44), while males accounted for 45.7% (n = 37). The average age of participants was 5.28 ± 1.93 years (Table 1).

The average follow-up period in this sample was 42.40 ± 21.51 months, with a minimum of 6 months and a maximum of 69 months (Table 2).

Of the total number of crowns included in the study (n = 167), a survival rate of 98.2% was observed. Three failed due to infectious processes, while 53 teeth had normal exfoliation, thus included in the clinical success rate (Table 3).

## 4. Discussion

The objective of this study was to evaluate the clinical success rate of treatments involving pulpotomies + IRM + PMC in primary teeth of children treated at a public university in Mexico, achieving a positive result in nearly 98.2% of cases. Intermediate Restorative Material (IRM) is one of the most used materials for performing pulpotomies; it is a biocompatible material that preserves pulp vitality and prevents leakage due to its sealing properties [22]. Although there are other material alternatives, our study included only pulpotomies with IRM that were restored with preformed metal crowns. The results are satisfactory when using these materials and could be implemented in situations where socioeconomic disadvantages are significant, thus providing treatments to remote and underserved communities.

The literature includes studies comparing the success of zinc oxide and eugenol with other materials, such as the study by Moskovitz et al. [38], which compared the efficacy of zinc oxide–zinc sulfate (Coltosol) versus zinc oxide–eugenol as filling material for the pulp chamber after applying formocresol to the surface of the radicular pulp. Results at 15.5 months of follow-up showed a success rate of 95% for Coltosol and 93.7% for zinc oxide and eugenol. At 45 months, the success rates were 79.7% for Coltosol and 67.7% for zinc oxide–eugenol. These results do not align with our study, which demonstrated greater success with follow-ups of up to 69 months, although these variations may be attributed to differences in methodology, as they based success on radiographic characteristics, which we could not consider in our study. On the other hand, the material used in our study was IRM, which, in addition to containing zinc oxide and eugenol, also includes polymers [38].

There are various parameters for measuring clinical success. In our study, we defined clinical success as the report of absence of pain, inflammation, and fistula or infection, like the study by Shumayrikh et al., which used the same parameters and achieved a success rate of 96.5%, also using zinc oxide and eugenol and the placement of preformed metal crowns as the final restoration [39].

To achieve a high clinical success rate in pulpotomy treatments, the final restoration is crucial for successful treatment [40]. This is why most of these treatments have been restored with metal crowns. In a systematic review by Randall et al., evaluating the efficacy of preformed metal crowns in primary molars, crowns demonstrated clinical success by being more durable and requiring less re-treatment [41]. This theory is supported by our study, as it coincides with the success we achieved in the treatments included that were restored with preformed metal crowns, showing follow-up periods of up to 69 months, maintaining the pulp tissue asymptomatic and demonstrating a success rate close to 100%.

Considering the clinical and epidemiological importance of these results, we can also highlight several points, considering the socioeconomic context of Mexico and its impact on oral health, which may apply to various countries. For example, the existence of socioeconomic inequalities and limited access to dental services. Mexico, like many developing countries, faces significant socioeconomic disparities that are reflected in access to oral health services [34,35]. The high prevalence of caries in the child population, especially in economically disadvantaged sectors, is a clear manifestation of these disparities. The limited coverage of free or accessible oral health programs contributes to many children not receiving the necessary care until oral diseases reach an advanced stage [36,37]. Similarly, the cost of dental materials in a country such as Mexico can be a limiting factor for providing services in public institutions. Thus, although there are advanced materials for pulpotomies that offer high success rates, many of these are expensive and not affordable for a large part of the Mexican population. Using more economical materials, such as Intermediate Restorative Material (IRM) and preformed metal crowns, which are affordable and effective, is crucial in this context [42,43]. This study demonstrates that, despite economic limitations, treatment with IRM and preformed metal crowns can achieve a high clinical success rate, making it a viable and cost-effective option, especially for public health institutions that could offer the service to underprivileged populations. Furthermore, in a country where a large portion of the population lives under unfavorable socioeconomic conditions, implementing affordable and effective treatments is fundamental to improving children’s oral health. Finally, the results of this study are particularly relevant for Mexico, where treating pulpotomies with IRM and preformed metal crowns can be an effective and sustainable solution to address the high rates of caries in children. Additionally, this approach offers an accessible and effective alternative for public health systems, which often face budgetary constraints. These findings underscore the need to promote oral health education, improve access to preventive and curative services, and prioritize cost-effective interventions in low-resource communities.

### 4.1. Future Research Directions

Future research should include a larger, prospective, randomized controlled trial comparing the pulpotomy + IRM + PMC approach to other commonly used pulpotomy techniques and restorative options in diverse populations. A more comprehensive cost-effectiveness analysis should be conducted to solidify the economic advantages of this methodology. Furthermore, long-term follow-up studies are essential to ascertain the longevity of clinical and radiographic success. Further research could also explore the effectiveness of this method in different socioeconomic contexts and investigate the use of alternative biocompatible materials for pulpotomy. Finally, incorporating detailed, standardized protocols for performing the procedure would increase reproducibility and enhance the quality of future studies.

### 4.2. Limitations

This study has limitations that must be considered for the proper interpretation of the data. One limitation was obtaining data indirectly; telephone questionnaires are a useful tool for data collection in a study of this type, as they provide an easy and straightforward way to gather information. However, some information bias may be introduced by the parents or caregivers of the children. On the other hand, during the study period, some of the children could not be located, which may introduce a selection bias. Another limitation is the ambispective design, reliance on parental recall for follow-up data, and the lack of a control group, which limits the generalizability of the findings. Moreover, the relatively short follow-up period (up to 69 months) may not fully capture long-term treatment outcomes. Selection bias may have been introduced by using children from a single public university setting.

## 5. Conclusions

This study demonstrates a high clinical success rate (98.2%) of treating pulpotomies in primary teeth with IRM and preformed metal crowns in a public university setting in Mexico. This cost-effective and accessible approach is particularly beneficial for children in rural and low-income communities with limited access to dental care. The biocompatibility of IRM, the durability of the crowns, and standardized training of clinicians appear to be key success factors. We recommend the implementation of this procedure in public health protocols and continued training in its application. However, large-scale prospective studies are needed to confirm these findings and assess their generalizability.

## Figures and Tables

**Table 1:** Distribution by sex and average age of the participants.

	**Frequency**	**Percentage**
Sex		
Girls	44	54.3
Boys	37	45.7
Total	81	100
	**Average ± SD**	**n**
Age	5.28 ± 1.93	81

**Table 2:** Average follow-up months for pulpotomies with IRM rehabilitated with stainless-steel crowns.

	Average ± SD	Min–Max
Follow-up Months	42.40 ± 21.51	6–69

**Table 3:** Clinical success rate of pulpotomies filled with IRM and restored with stainless-steel crowns.

	Frequency	Percentage
Treatment Efficacy		
Clinical Success	164	98.2
Failure: Infectious Process	3	1.8
Total	167	100

## Data Availability

The datasets generated and/or analyzed during the current study are available from the corresponding authors upon reasonable request.
